# Calcium Hydroxylapatite Combined with CO_2_ Ablative Fractional Laser for Atrophic Acne Scars: A Randomized Controlled Study

**DOI:** 10.1007/s00266-026-06142-1

**Published:** 2026-07-06

**Authors:** Weiliang Chen, Liyuan Zhang, Xiao Luo, Chenxi Zhou, Kai Li, Xiaolei Qin

**Affiliations:** 1 Deyi Skin, Dexi Medical Aesthetic Clinic, Xi’an, Shaanxi China; 2 Deyi Skin, Deyue Medical Aesthetic Clinic, Shenzhen, Guangdong China

**Keywords:** Calcium hydroxylapatite (CaHA), CO_2_ ablative fractional laser (AFL), Atrophic acne scars

## Abstract

**Background:**

This study was to investigate the efficacy and safety of calcium hydroxylapatite (CaHA) filler injections combined with CO_2_ ablative fractional laser (AFL) for atrophic acne scars.

**Methods:**

A prospective, single-center, randomized controlled study was conducted on 160 participants, who were average randomly assigned to either an observation group (A) or a control group (B). Group A received two sessions of CaHA (1-month interval) after AFL 1 month, while Group B received AFL monotherapy. Efficacy was assessed by pre- and post-operation of standardized photographic images. Additionally, treatment-related adverse events (AEs) were documented.

**Results:**

Compared with baseline, the quantitative global scarring grading system scores decreased significantly in both groups (*p* < 0.001), with greater reduction observed in group A (*p* < 0.001). The acne scar rating scale analysis revealed the combination therapy’s effectiveness across all scar subtypes (*p* < 0.001) and demonstrated comparable efficacy for boxcar and rolling scars (*p* = 1.000), both of which showed superiority to icepick scars (*p* < 0.001). Mild AEs are occurred in both group, such as pain, edema, crusting, and transient post-inflammatory erythema (PIE), CaHA injection may pose a risk of ecchymosis. Nevertheless, long-term PIE (lasting over 4 weeks) (*p* < 0.001) and post-inflammatory hyperpigmentation (*p* = 0.002) in group A exhibited significantly shorter healing times, accelerated healing by an average of 3.8 weeks and 3.7 weeks.

**Conclusion:**

The combination of CaHA with AFL demonstrates both efficacy and safety for atrophic acne scars, particularly boxcar and rolling subtypes.

**Level of Evidence II:**

This journal requires that authors assign a level of evidence to each article. For a full description of these Evidence-Based Medicine ratings, please refer to the Table of Contents or the online Instructions to Authors www.springer.com/00266.

## Introduction

Acne vulgaris, a prevalent chronic inflammatory disease of the pilosebaceous unit, results in scarring in approximately 95% of affected individuals globally [1]. These scars exert a significantly negative psychological impact. Epidemiologically, atrophic scars demonstrate a 3:1 prevalence ratio compared to hypertrophic and keloid scars combined [1]. Morphologically, the classification system delineates three atrophic scar subtypes: icepick, boxcar, and rolling (Table 1).

**Table 1:** Atrophic acne scar morphologies

Scar subtypes	Description
Icepick	Narrow indentations that demonstrate tapered effect from the surface to deepest aspect, resembling that of an icepick, “V” shaped, diameter < 2 mm.
Boxcar	Round or oval depressions with sharp, vertical edges, “U” shaped, vary between 1.5 and 4 mm wide, and further subdivided as shallow (0.1–0.5 mm) or deep (≥ 0.5 mm).
Rolling	Shallow, widened depression caused by intermittent tethering of the dermis to the subcutis subcutis creating shadows that produce a “rolling” appearance, sloped edges that merge with normal appearing skin, “M” shaped, diameter (4–5 mm).

The CO_2_ ablative fractional laser (AFL) is a widely preferred energy-based device (EBD) for scars management. Nevertheless, the requirement for multiple sessions not only prolongs course of treatment but heightens the risk of adverse events (AEs), such as post-inflammatory erythema (PIE) and hyperpigmentation (PIH), particularly with darker skin tones, where AFL monotherapy demonstrates suboptimal outcomes for rolling and icepick scars [2, 3].

Contrastly, biomaterial-based filler therapies are emerging as viable alternatives for them. Current options include hyaluronic acid (HA), calcium hydroxylapatite (CaHA), and poly-L-lactic acid [4]. CaHA has demonstrated unique therapeutic advantages in stimulating regeneration of type I or III collagen, elastin, and proteoglycans, while also repairing the vasculature of tissues [5], though randomized controlled trials evaluating its efficacy and safety profile in this specific application remain limited.

This study employed a prospective, single-center, randomized controlled trial design to investigate the therapeutic efficacy and safety profile of CaHA combined with AFL for atrophic acne scars, with the goal of addressing the inherent limitations of monotherapy approaches.

## Materials and Methods

### Design and Participants

A total of 160 subjects participated in this trial conducted at Dexi medical aesthetic clinic Deyi Skin from December 2023 to March 2025. Participants were averagely allocated to observation group (A) or a control group (B) via a random number generation method.

Patients meeting the following criteria were included in the study: (1) aged 18–60 years; (2) clinical diagnosis conforms to atrophic acne scars on the face; and (3) ability to comply with treatment and follow-up, with complete data meeting study protocol requirements.

Exclusion criteria: (1) any facial cosmetic interventions within less than 3 months pre-enrollment, including chemical peels, EBD treatments, therapeutic regimens for scars (systemic retinoids/topical steroids); (2) history of facial injection filler treatments within 6 months prior to the study; (3) pregnant or lactating women; (4) individuals allergic to study agents or with photosensitivity; (5) individuals with tendency of keloids or hypertrophic scars (scarring diathesis); and (6) systemic comorbidities: autoimmune diseases, metabolic (uncontrolled diabetes, thyroid dysfunction), neuropsychiatric, cardiovascular, hepatopathy, or nephrosis.

## Treatment Protocol

Group A received two sessions of CaHA (1-month interval) after AFL 1 month, while group B received AFL monotherapy. The clinical observation endpoint is 6 months after AFL.

**Fig. 1:**
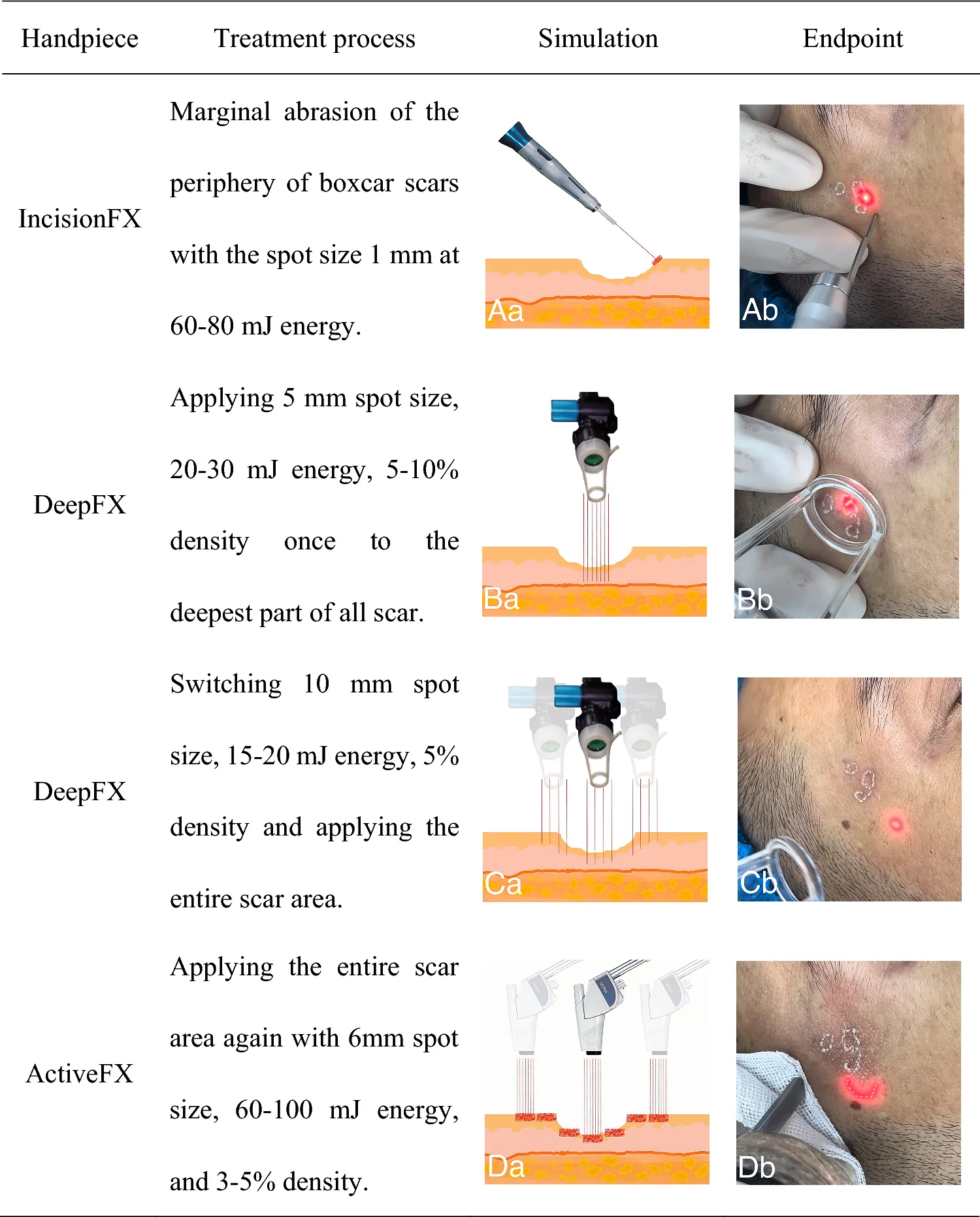
The AFL treatment process

All subjects underwent a standardized AFL procedure under topical anesthesia with 5% compound lidocaine cream (2.5% lidocaine + 2.5% prilocaine) applied under occlusion for 1 hour pre-operatively. A CO_2_ surgical laser system (UltraPulse Encore, Lumenis Be Ltd., United States of America) was utilized in which treatment process were as follows Fig. 1. The ultrapulse duration was 250 us. Intraoperative management included dynamic cooling (Zimmer Cryo 6, Zimmer MedizinSysteme GmbH, Germany) titrated to maintain subject comfort during laser-tissue interaction. Post-operative care required closed moist wound healing until complete re-epithelialization. Subjects were mandated to: (1) strictly avoid UV exposure 3 months; (2) adhere to mineral or physical sun protection outdoors; and (3) eliminate photosensitizing dietary components.

**Fig. 2:**
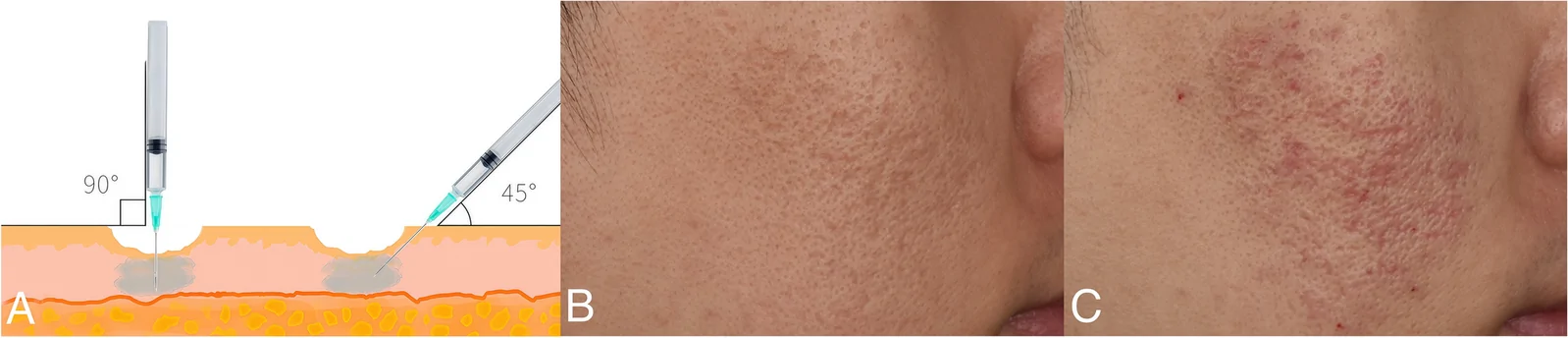
CaHA injection treatment. **A**, Injection simulation; **B**, Pre-injection; **C**, Post-injection immediately

Additionally, the injection protocol utilized 50 mg lyophilized CaHA powder (HA/F 125-90-74; Sichuan Baiamon Bioactive Materials Co., Ltd., China) reconstituted with 2.5 ml sterile saline 0.5 ml 2% lidocaine HCl and 2 ml non-crosslinked HA gel (10 mg/mL; Changzhou Institute of Materia Medica Co., Ltd., China). Aseptic compounding was achieved via vortex mixing through a Luer-lock 3-way stopcock, yielding a homogenous 5 ml suspension. Dosage was titrated (3–5 mL/session) based on extent and severity of atrophic scars. A 30G 4-mm injection needle was inserted vertically or at 45° into the deep dermis and subcutaneous soft tissue defects, with a single-point dose not exceeding 0.05 ml (Fig. 2A). The injection endpoint was defined as concave scar areas becoming flush with or slightly raised above the surrounding normal skin (Fig. 2B, C). Immediately post-procedure, the treating physician gently massaged the area until the skin was smooth. For any persistent raised areas within 3–5 days, subjects were instructed to self-massage for 5–10 min daily.

## Measurements

### Digital Photography

Prior to treatment, all subjects underwent a comprehensive facial skin evaluation after cleansing. Standardized multi-angle facial photographs (frontal, left, and right lateral 45°) were taken using a multispectral imaging analyzer (ThinkView, Wuhan Bose Electronic Co., Ltd., China) before each treatment and endpoint by blinded assessors. These photos were systematically archived for subsequent assessment.

### Efficacy Assessment

Two blinded assessors used the quantitative global scarring grading system (GSS) to rate based on pre- and post-operation condition [6]. To assess efficacy on different scar subtypes, the 4-point acne scar rating scale (ASRS) was used (1 = minimal, 2 = mild, 3 = moderate, 4 = severe) to score baseline and post-procedural in group A [7]. Their average score was statistically analyzed, and △GSS or △ASRS was defined as the difference between pre- and post-treatment scores.

Based on the last follow-up compared to pre-operation, independent physicians evaluated overall visual improvement using a 4-point global assessment score (GAS): 0 = no improvement or worsening; 1 = marginal improvement (<25%); 2 = mild improvement (25–<50%); 3 = moderate improvement (50–<75%); 4 = marked improvement (≥75%). All participants completed a satisfaction survey rating their improvement (0 = dissatisfied; 1 = neutral; 2 = minimally satisfied; 3 = satisfied; 4 = highly satisfied).

### Safety Assessment

Pain severity following each treatment was assessed using the numeric rating scale (NRS, 0 = no pain, to 10 = worst imaginable pain). All other treatment-related AEs were documented. Protocol mandated immediate medical consultation for severe AEs or urgent clinical deterioration.

### Statistical Analysis

Statistical analysis was performed using SPSS 26.0 (IBM Corp., United States of America). Descriptive statistics were presented as mean ± standard deviation (SD) and percentages (%). The Chi-square test was used for categorical data. Student’s unpaired t-test or paired t-test was used for normally distributed continuous variables, and Mann–Whitney U test was applied to non-normally distributed continuous variables. Intragroup differences were analyzed using the Wilcoxon signed-rank test. One-way ANOVA with post hoc tests was used for multi-group comparisons. Statistical significance was set at *p* < 0.05 (two-tailed).

## Results

### Baseline Characteristics

A participant from group B was lost to follow-up due to relocation and was therefore excluded from the final analysis. Another 159 subjects were completed the trial, including 42 (26.4%) males and 117 (73.6%) females. Ages ranged 19-46 years (mean 29.11 years). Scar duration ranged 1–21 years (mean 9.75 years). Pre-GSS scores ranged 10-38 points (mean 23.13 points). As shown in Table 2, no statistically significant differences in baseline between the two groups (*p* > 0.05).

**Table 2:** Baseline characteristics of participants

		n (%) or Mean ± SD		t or Z	χ^2^	*p*-value
		Group A (n = 80)	Group B (n = 79)			
Gender	Male	18(22.5)	24(30.4)	–	1.270	0.260
	Female	62(77.5)	55(69.6)			
Fitzpatrick type	II	8(10.0)	6(7.6)	–	0.389	0.823
	III	53(66.2)	52(65.8)			
	IV	19(23.8)	21(26.6)			
Age (years)		29.40 ± 4.24	28.82 ± 4.55	0.827	–	0.410
Duration (years)		10.06 ± 4.28	9.44 ± 4.68	0.871	–	0.385
GSS (points)		23.68 ± 6.96	22.56 ± 5.60	1.116	–	0.266

## Efficacy Assessment

**Fig. 3:**
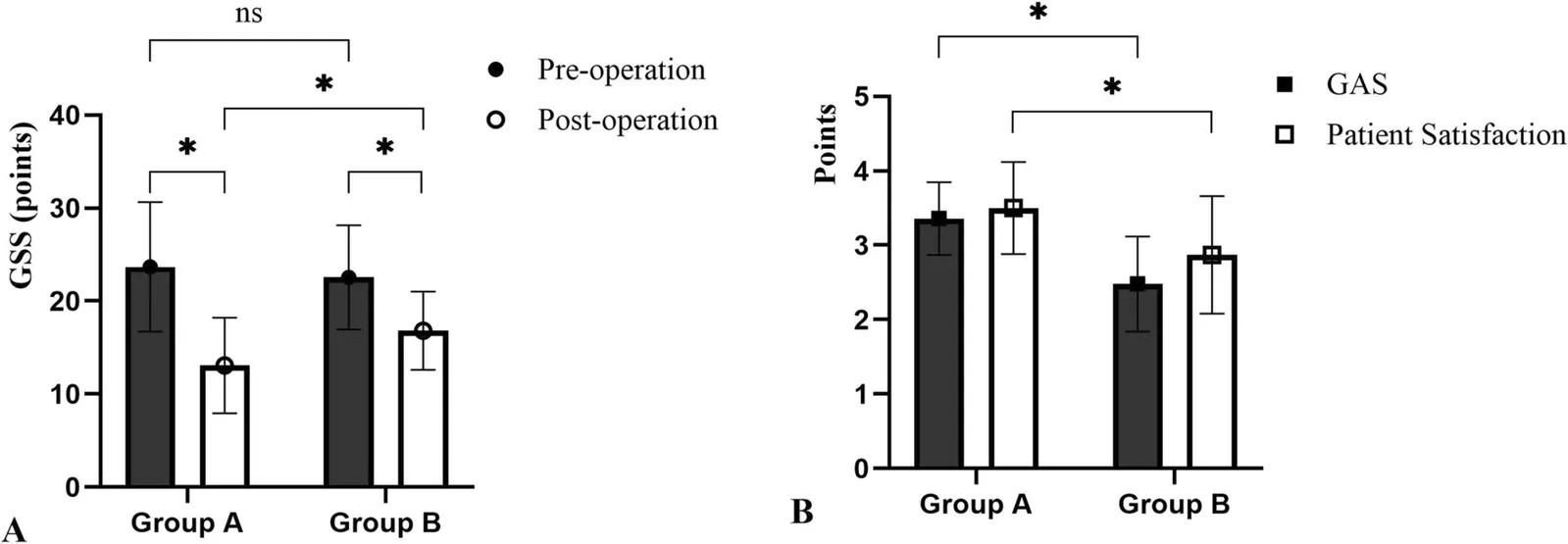
Efficacy between the two group. ns, *p* > 0.05; *, *p* < 0.001

As depicted in Fig. 3A, GSS scores showed significantly descension in both groups compared to baseline (group A: t = 12.648, *p* < 0.001; group B: t = 17.181, *p* < 0.001). Furthermore, group A exhibited significantly greater ΔGSS (group A: 10.61 ± 7.50 points; group B: 5.74 ± 2.97 points; t = 5.029, *p* < 0.001). In Fig. 3B, both the GAS assessed by blinded assessors (group A: 3.36 ± 0.49 points; group B: 2.48 ± 0.64 points; t = 9.763, *p* < 0.001) and the patient satisfaction (group A: 3.50 ± 0.62 points; group B: 2.87 ± 0.79 points, t = 5.578, *p* < 0.001) consistently demonstrated group A’s superiority over group B with statistical significance.

**Fig. 4:**
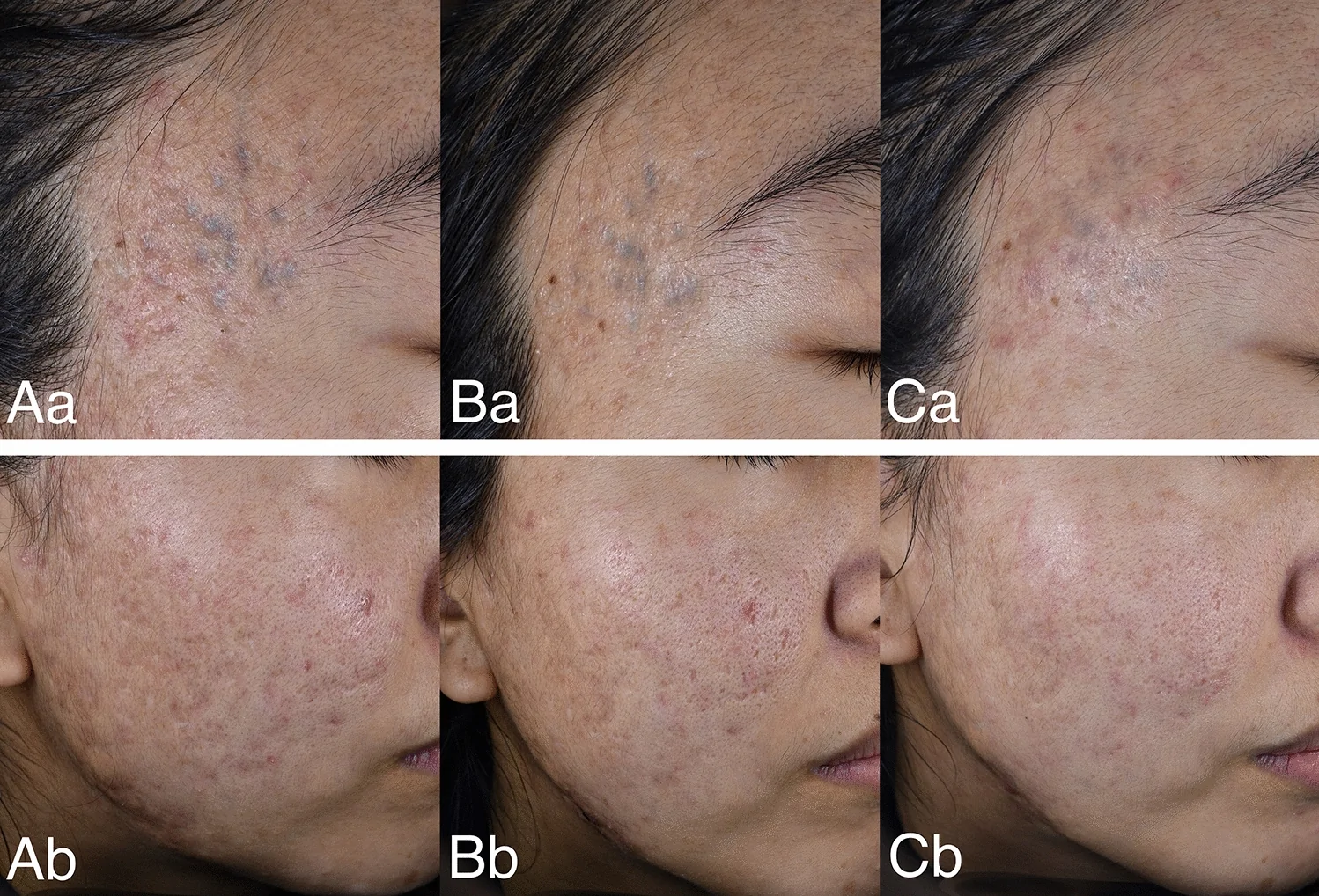
A 26-year-old female received combination therapy in group A. Aa/Ab, Pre-operation; Ba/Bb, Post-operation; Ca/Cb, Post-operation after 1 year

To further investigate the combined therapy efficacy on different scar subtypes, the results are shown in Table 3, paired t-tests validated treatment efficacy for all subtypes (all *p* < 0.001). Post hoc pairwise comparisons indicated comparable efficacy between boxcar and rolling scars (*p* = 1.000), both showing superiority over icepick type (*p* < 0.001). Fig. 4 clearly shows the improvement of various subtype in temporal and cheek of a 26-year-old female after combined treatment.

**Table 3:** Improvement in atrophic scar subtype based on combination therapy

Subtypes	Cases (n)	ASRS (Mean ± SD)			t	*p*-value
		Pre-operation	Post-operation	△ASRS		
Icepick	74	2.63 ± 0.95	2.20 ± 0.79	0.43 ± 0.42	8.731	< 0.001
Boxcar	80	2.84 ± 0.60	1.72 ± 0.64^a^	1.13 ± 0.42	23.692	< 0.001
Rolling	75	2.66 ± 0.89	1.73 ± 0.64^ab^	0.93 ± 0.47	17.293	< 0.001
F		1.932	10.383	51.856		
*p*-value		0.149	< 0.001	< 0.001		

^a^, versus Icepick, *p* < 0.001; ^b^, versus Boxcar, *p* = 1.000.

## Safety Assessment

**Fig. 5:**
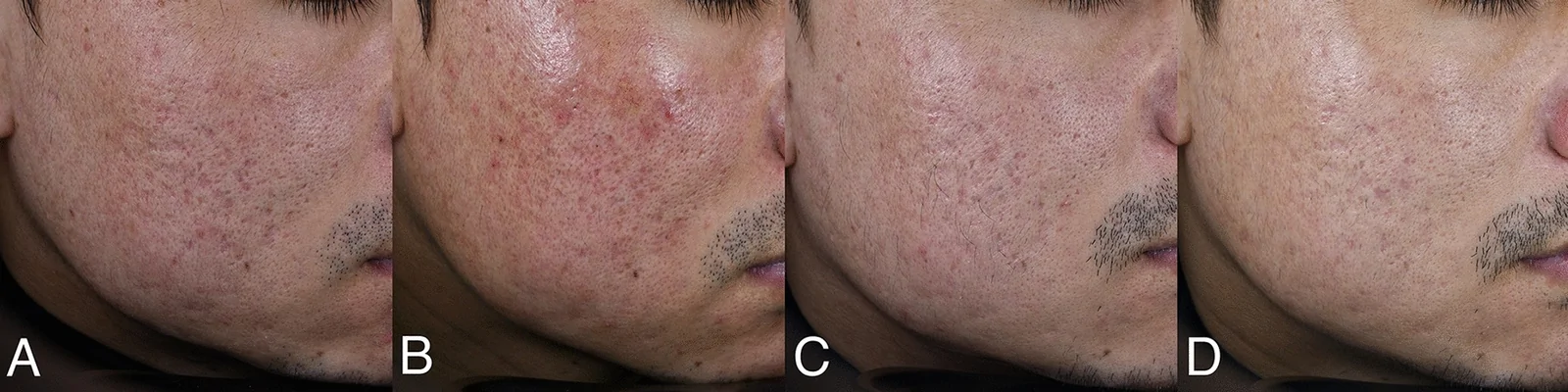
A 31-year-old male received combination therapy in group A. **A**: Pre-operation; **B**, AFL after 1 month; **C**, Post-operation; **D**, Post-operation after 1-year

AEs revealed universal occurrence of edema, PIE, and pain during treatment and throughout the 48h post-operative period. Edema and pain generally resolved spontaneously within 24–72h. Over half of the transient PIE demonstrated complete resolution within 1–4 weeks. The NRS scores (group A: 5.81 ± 1.15 point; group B: 5.62 ± 1.18 point) showed comparable intensity (t = 1.041, *p* = 0.299). Despite the recommendation to moist wound healing, all subjects experienced crusting, which desquamated within 5–10 days. In Table 4, the incidence of long-term PIE (lasting over 4 weeks) and PIH did not differ significantly between the groups (all *p* > 0.05). Nevertheless, the healing durations of PIE (*p* < 0.001) and PIH (*p* = 0.002) were significantly shorter in group A, accelerated healing by an average of 3.8 weeks and 3.7 weeks. As shown in Fig. 5, occurring in PIE and PIH after AFL 1-month (5B), the patient was relieved after injection treatment (5C). Furthermore, post-injection ecchymosis was occurred in 28 (35.0%) cases of group A, resolving without intervention within 1–2 weeks. Until the endpoint of clinical observation follow-up, no persistent AEs such as skin nodules, granulomas, tissue overgrowth, or chronic inflammation were observed.

**Table 4:** Comparison of long-term PIE and PIH between in two groups

AEs		n (%) or Mean ± SD		χ^2^ or t	*p*-value
		Group A	Group B		
Long-term	Cases (n)	35 (43.8%)	32 (40.5%)	0.172	0.679
PIE	Duration (weeks)	7.23 ± 1.54	11.00 ± 2.45	7.470	< 0.001
PIH	Cases (n)	16 (20.0%)	18 (22.8%)	0.183	0.668
	Duration (weeks)	10.38 ± 1.96	14.06 ± 3.95	3.499	0.002

## Discussion

In this study, the average age was 29 years, with female predominance (73.6%), reflecting the substantial clinical demand for acne scar improvement among this population. The mean scar chronicity of 9.75 years underscores the persistent disfigurement burden in this population.

The pathogenesis of atrophic acne scars is multifactorial and incompletely characterized. Current consensus posits inflammatory acne lesions induce perifollicular abscess rupture, triggering peri-inflammatory cascades and subsequent pathologic healing response [1]. An elevated ratio of matrix metalloproteinase (MMPs) to tissue inhibitors of tissue inhibitor of matrix metalloproteinases (TIMPs) weakens collagen synthesis during extracellular matrix (ECM) remodeling [4, 8]. MMP-2-mediated subcutaneous adipose tissue occurs concomitantly with periadnexal fibrotic changes [4]. Histopathologically, changes include epidermal structural alterations, pilosebaceous unit loss in the dermis, inflammatory cell infiltration, varying degrees of insufficient dense collagen fiber deposition, decrease in elastic fiber, contracture [9]. Type I or III collagens impart the most structure and rigidity, which act as major cellular scaffolds. The ratio of them and their degree of crosslinking largely determine the skin’s tensile and mechanical strengths [5]. Furthermore, our previous research indicates therapies targeting blood vessels, which repair the tissue vascular system, can also effectively alleviate acne scars [10]. Therefore, targeted disrupting altered post-inflammatory microarchitecture and promoting orderly regeneration of cellular and ECM components constitute therapeutic imperatives.

Currently, AFL remains the preferred EBD for improving acne scars management, operating at 10600 nm wavelength with preferential absorption by intra/extracellular water. Based on the principles of selective photothermolysis [11] and fractional photothermolysis [12], laser beams create dense, uniformly arranged columnar microthermal zones (MTZs) and surrounding thermal coagulation zones, initiating a programmed wound healing process. The efficacy and AEs observed in Group B align with previous studies [13–16], in which edematous erythema and pain manifested universally. Despite continuous epidermal cooling, the average NRS score reached 5.62 points (total 10 points). Various measures have been explored to reduce pain during AFL treatment, such as topical 30% salicylic acid [16], locally applied vibrating devices [17], and intravenous dezocine [18]. For individuals with darker skin tones, AFL carries higher risk of long-term PIE and PIH, sometimes lasting over 3 months [14, 15]. Although equivalent efficacy has been reported with 1-month versus 3-month AFL intervals [19], in practice, most adjustments to the treatment regimen cycle have to be delayed due to unresolved mid- and long-term AEs. Moreover, limited patient compliance stems from prolonged post-procedure downtime. Thus, combined treatment regimens offer an effective strategy to shorten the total treatment duration [4], synergistically enhance scar remodeling and mitigate AEs.

Soft tissue augmentation has emerged as an acceptable therapeutic modality for atrophic acne scars, particularly in cases exacerbated by cutaneous laxity or volume loss in the cheek or chin area, as a deflated balloon, which can worsen in appearance with age or photodamage [3, 20]. The CaHA has a porous microsphere structure, with the CaHA spheres utilized in this study having a diameter of 25–40 µm and porosity exceeding 60%. In previous studies, CaHA is usually suspended in viscoelastic crosslinked HA and remains positionally stable at the target site after implantation [21]. Simultaneously, the supportive HA temporarily alleviates downward traction from fibrotic subcutaneous tissue, and also, its elastic modulus provides immediate filling effects to compensate for soft-tissue deficits within atrophic scars improving appearance. Nevertheless, to minimize HA-related confounding and more directly assess CaHA’s effect on scar tissue, we employed non-crosslinked HA solely as a short-term vehicle for the uniform suspension of CaHA microspheres. As HA is absorbed in the short term due to its non-crosslinked nature, CaHA microspheres gradually enveloped by a network of collagen fibers, fibroblasts, and macrophages, initiating cell-biomateria mediated interaction [5]. The sustained positional stability of CaHA microspheres, if any, likely depends on subsequent tissue integration rather than the transient presence of HA. This serves as a scaffold for fibrous proliferation and new collagen formation, gradually achieving endogenous type I or III collagen and elastin regeneration. Recent studies demonstrate that CaHA enhances fibroblast function and induces normal collagen fibers by upregulating calcium signaling and motor protein signaling pathways, promoting ECM maturation and skin structural integrity reconstruction [22]. Despite the use of non-crosslinked HA, its effect cannot be considered negligible. Consequently, our study is limited by the inability to separately ascertain the short-term benefits derived from CaHA versus HA.

A retrospective study revealed superior outcomes when CaHA pretreatment preceded AFL (1-month interval) compared to AFL monotherapy [22]. It is regretful that this prior investigation neither explored CaHA injection administration post-AFL, nor elucidated the pathophysiological rationale for treatment sequence optimization. We hypothesize that injecting CaHA first, even with a long interval before AFL, might risk deactivation or compromise the collagen-forming environment due to subsequent vaporization and thermal stress from AFL. While the optimal treatment sequence warrants further investigation, our study similarly supports that CaHA enhances AFL efficacy for acne scars. The combined therapy proved superior across multiple metrics including △GSS, GAS and patient satisfaction survey. Some subjects (such as Fig. 4 and Fig. 5) continue to be followed up after the endpoint of clinical observation, although no further treatment was administered during post-operation after 1-year, we still come across the scars are persistently improving.

Furthermore, while previous studies emphasized AFL is primarily effective for boxcar scars with less impressive results for other subtypes [2, 3], our findings demonstrate combined therapy effective for all subtypes (*p* < 0.001). Notably, ASRS improvement for boxcar scars (1.13 ± 0.42 points) was numerically higher than for rolling scars (0.93 ± 0.47 points), but there was no significant difference in efficacy between them (*p* = 1.000). This result is similar to previous findings [8]. Some scholars believe that it is significant to inject dermal filler into the dense fibrotic areas to relax the collagen fiber arrangement [9]. CaHA injection may optimize efficacy for rolling scars. Pathophysiologically, rolling scars are characterized as scar bands extending from the dermis to the subcutaneous tissue resulting in dimpling of the skin. We believe that the volumizing effect and needling response of CaHA precisely injection may achieve a certain degree of dense tissue release, while addressing the treatment blind spot left by low-fluence AFL that cannot reach the target tissue. AFL treatment may result in PIE or PIH that cannot be completely healed in the short term. An unexpected benefit observed was the accelerated alleviation of them after combined injection. The CaHA modulates inflammatory responses, restores vascular activity, promotes pigment metabolism, and accelerates re-epithelialization for skin repair [23]. Despite the admixture of non-crosslinked HA during injection, CaHA may exert a greater influence on medium- to long-term PIE and PIH. Although injection therapy increases the risk of ecchymosis, for aesthetic, according to the author’s experience, immediately combining a dye laser would accelerate restoration, typically within 5–7 days.

Ideal soft-tissue fillers exhibit well biocompatibility, biodegradability, low immunogenicity, and minimal displacement. CaHA, a major inorganic component naturally present in the human body, is immuno-inert with minimal adaptive immune cell recruitment and without chronic inflammation [5]. Histological study was observed that no inflammatory response to the CaHA filler after 1-month and 6-month, although bits of macrophages and foreign body giant cells were detected. While the CaHA was homeostatically degraded, macrophage mediated phagocytosis was the primary clearance mechanism [24], a process potentially related to microsphere size [25]. Nevertheless, persistent AEs warrant attention. A real-world study reported 3% cases AEs consisted of 96% cases nodules with 45% occurring in dynamic areas [26]. In our study, until the endpoint of clinical observation follow-up, no persistent AEs were observed. Furthermore, 69 (86.3%) participants are still under follow-up observation or receiving other treatments, but nodules, granulomas, tissue overgrowth, or chronic inflammation have not been found even after 1-year of CaHA injection. While some research suggests CaHA stimulates collagen production lasting up to 18 months [27], and magnetic resonance imaging studies indicate CaHA can completely disappear within 30 months [28], a limitation of our study is the need for longer follow-up to more accurately ascertain long-term safety.

## Conclusion

This prospective, single-center, randomized controlled trial demonstrates that combined CaHA filler injection and AFL are an effective and safe multimodal intervention for atrophic acne scars, demonstrating optimal results for boxcar and rolling types. The combination regimen not only synergistically enhances scar remodeling outcomes, but CaHA injection therapy significantly promotes post-AFL recovery by mitigating AEs.
